# Persistent Afferent Bias: a mechanistic link between somatic disruption and autonomic dysregulation

**DOI:** 10.3389/fnint.2026.1848233

**Published:** 2026-07-03

**Authors:** Boris Živný

**Affiliations:** NeuroCentrum Clinic, SPP Institute, Jesenice, Czechia

**Keywords:** Persistent Afferent Bias, Somato-Psychic Pathway, Somato-Psychic Autonomic Dysregulation, afferent weighting, autonomic dysregulation, neural regulation, neurodevelopmental disorders, somatic disruption

## Abstract

Persistent Afferent Bias (PAB) represents a state-dependent alteration in sensory weighting through which Somatic Disruption influences Autonomic Regulation. While the classical concept of the facilitated spinal segment provided an early neurophysiological explanation for somatic dysfunction, contemporary neuroscience increasingly favors models based on distributed neural networks and dynamic modulation of neural gain. Recent reinterpretations of facilitation as a network-level phenomenon offer a more consistent account of altered sensorimotor processing, yet remain largely confined to musculoskeletal and pain-related mechanisms and do not fully explain the origin and persistence of such bias. The present paper proposes Persistent Afferent Bias as a mechanistic link between Somatic Disruption and Autonomic Dysregulation. Within the Somato-Psychic Pathway (SPP), a theoretical model proposed by the author, PAB represents the previously unarticulated neurophysiological mechanism linking altered somatic input to changes in regulatory state. In this context, disturbances in afferent signaling—arising from Distorted Proprioceptive Information (DPI), nociceptive input, interoceptive imbalance, or other sources—lead to a reorganization of sensory weighting within neural systems. A defining feature of this process is the persistence of altered afferent conditions. Under sustained conditions, this biased processing contributes to a shift in Autonomic Regulation conceptualized as Somato-Psychic Autonomic Dysregulation (SPAD), which may subsequently give rise to broader functional manifestations, including Somato-Psychic Syndrome (SPS). This formulation situates Persistent Afferent Bias within a hierarchical regulatory cascade consistent with Directional Developmental Sequencing (DDS), in which alterations at the level of somatic input propagate to autonomic and higher-order functional domains. In this sense, PAB is not a primary phenomenon, but a secondary expression of underlying disturbances within the somatic regulatory system. Beyond its theoretical significance, this model has direct clinical implications. It supports a shift in clinical reasoning from predominantly symptomatic modulation toward identification and targeted treatment of primary somatic drivers of altered afferent input. This is particularly relevant in patients presenting with Somato-Psychic Syndrome (SPS), in whom conventional approaches focused on higher-order manifestations may fail to address the underlying regulatory disturbance. By providing a mechanistic basis for this shift, Persistent Afferent Bias enables a more etiologically grounded and hierarchically organized approach to intervention within the Somato-Psychic Pathway.

## 1 Historical background: the facilitated segment

The concept of bias has emerged in contemporary neuroscience as a fundamental property of neural systems, reflecting the dynamic integration of prior states and incoming sensory input. Rather than representing a failure of processing, bias increasingly appears to constitute an organized and functionally meaningful aspect of neural regulation. Within this broader context, the concept of the facilitated spinal segment has historically served as one of the central theoretical explanations for somatic dysfunction in osteopathic medicine. In the model proposed by (Korr, 1947; Denslow and Korr, 1947), sustained afferent input from somatic tissues was believed to lower neuronal firing thresholds in specific spinal segments, thereby amplifying motor, sensory, and autonomic outputs.

This model provided an early neurophysiological framework to explain clinical observations such as segmental tenderness, altered muscle tone, reflex guarding, and viscerosomatic patterns. These phenomena were interpreted as evidence of persistent segmental facilitation.

However, advances in neurophysiology suggest that such persistent hyperexcitability is unlikely to remain confined to isolated spinal segments in the absence of structural pathology. This has led to growing efforts to reinterpret osteopathic concepts in light of modern neuroscience.

## 2 Contemporary reinterpretation: Persistent Afferent Bias

Contemporary neuroscience increasingly recognizes that bias is not merely a byproduct of imperfect processing but a fundamental property of neural systems reflecting the dynamic integration of prior states and incoming sensory input. This is supported by recent evidence demonstrating that such biases are associated with identifiable neural signatures and organized patterns of processing rather than random distortions (Geisler and Meyer, 2025). At the same time, attentional bias appears to be dynamically regulated and context-dependent, reflecting an active neurophysiological process rather than a passive distortion (Pinto et al., 2026). Within this framework, alterations in sensorimotor processing are conceptualized as state-dependent changes in the relative weighting of afferent input within distributed neural networks.

In this context, neurons remain structurally and functionally intact, while the influence of specific afferent streams on neural processing is altered. Certain inputs may exert a disproportionate effect on system behavior, resulting in changes in neural gain, reflex responsiveness, and autonomic output.

Such Persistent Afferent Bias may arise from multiple sources, including nociceptive input, muscle spindle activity, fascial mechanoreceptor signaling, and viscerosomatic convergence. These influences do not reflect localized structural pathology, but rather a dynamic reorganization of sensory weighting within integrated sensorimotor systems (Woolf, 2011; Fields, 2004).

This reinterpretation shifts the concept of facilitation from a segmental model of persistent neuronal hyperexcitability to a network-based model of state-dependent neural regulation. However, while this perspective explains how afferent bias operates within neural systems, it does not fully account for why such bias emerges or persists over time.

## 3 State-dependent neural regulation

Modern neuroscience increasingly recognizes that neuronal responsiveness is shaped not only by structural connectivity but also by the system’s current physiological state. This state reflects a dynamic integration of autonomic tone, neuromodulatory activity, metabolic and inflammatory signals, and descending regulatory influences. In this context, bias may be understood as an intrinsic property of this state-dependent organization that emerges from the continuous interaction between prior system states and incoming afferent input.

Within this framework, sensory processing is not a passive reflection of incoming input but an actively regulated process. Contemporary models describe this regulation as predictive, in which the nervous system continuously integrates afferent input with prior expectations to optimize adaptive responses (Barrett and Simmons, 2015; Friston, 2010). Within this framework, alterations in afferent weighting represent a specific mechanism through which such predictive and state-dependent dynamics are expressed, linking changes in somatic input to broader shifts in neural and autonomic regulation.

As a consequence, identical sensory inputs may produce markedly different physiological and behavioral outputs depending on the system’s regulatory state. This principle provides a mechanistic explanation for clinical observations such as variability in tenderness, context-dependent reflex responses, and the rapid reversibility of somatic findings (Fields, 2004; Friston, 2010).

Recent research further demonstrates that persistent nociceptive input can bias attentional and perceptual processing, leading to preferential weighting of pain-related information (Li et al., 2025). In addition, attentional bias itself appears to be dynamically regulated and context-dependent, reflecting an active neurophysiological process rather than a passive distortion (Pinto et al., 2026). These findings support the broader principle that sustained afferent input can systematically alter neural gain and information processing.

More generally, recent work suggests that perceptual and decision-related representations are continuously shaped by the system’s prior states, with past activity reactivated and interacting with ongoing processing in a temporally structured manner (Luo et al., 2025). At the same time, other findings indicate that certain forms of bias may reflect more stable organizational tendencies within neural systems, such as preferential allocation of processing resources toward self-relevant information (Geisler and Meyer, 2025).

Taken together, these observations suggest that bias is neither a fixed defect nor a transient disturbance, but an emergent property of neural systems that is both dynamically regulated and structurally constrained. In this context, alterations in afferent weighting should be understood not as errors or distortions but as state-dependent regulatory processes that reflect ongoing adjustments within distributed neural systems.

This perspective is broadly consistent with contemporary models of autonomic organization and behavioral regulation, which emphasize the dynamic coupling between physiological state and adaptive responses (Porges, 2025).

Distorted proprioceptive information (DPI), Persistent Afferent Bias (PAB), and Somato-Psychic Autonomic Dysregulation (SPAD) may be understood as three functionally distinct yet hierarchically related levels within a single regulatory cascade. DPI refers to an alteration in the quality of incoming sensory input, typically arising from disturbances within the somatic system. While DPI often represents a clinically prominent source of altered afferent input, it constitutes only one of several potential contributors to the emergence of PAB. Other sources may include persistent nociceptive input, altered muscle spindle activity, visceral afferent signaling, or changes in interoceptive processing, among other potential contributors not further elaborated here (Craig, 2009; Friston, 2010; Barrett and Simmons, 2015; Seth and Friston, 2016). PAB represents a state-dependent reorganization of sensory weighting within neural networks, in which certain afferent streams exert a disproportionate influence on system behavior. SPAD, in turn, reflects a more stable alteration in autonomic regulation, emerging when such biased processing becomes functionally embedded within the organism’s regulatory state. Within this framework, DPI does not directly constitute dysregulation, but contributes to its emergence by shaping the afferent conditions under which the system operates. In this context, PAB may be viewed as a transitional mechanism linking somatic disruption to autonomic dysregulation.

**Figure 1:**
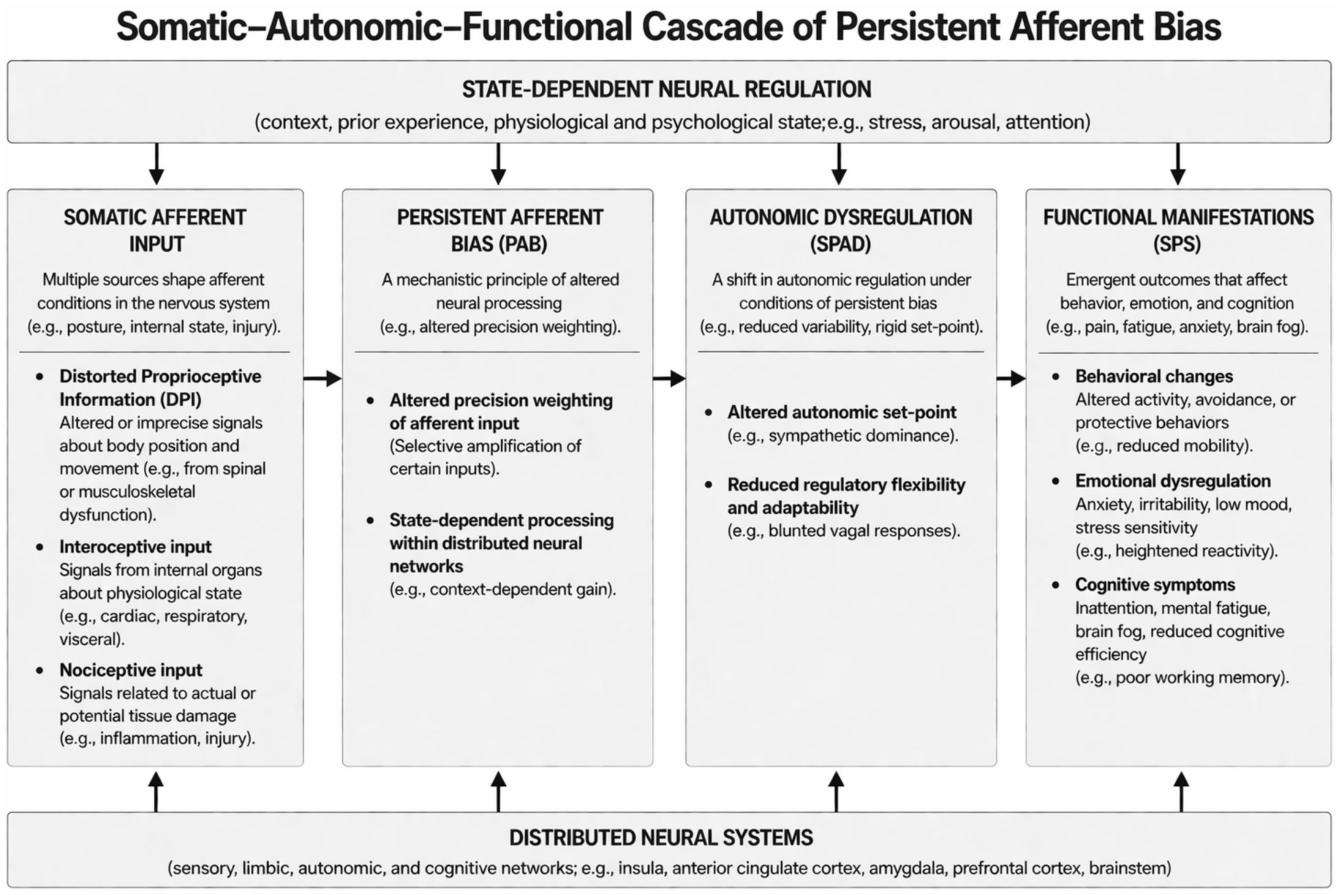
Somatic–autonomic–functional cascade of Persistent Afferent Bias. The diagram illustrates the proposed hierarchical relationship between somatic input, neural processing, autonomic regulation, and functional outcomes. Somatic afferent input, including Distorted Proprioceptive Information (DPI), interoceptive signals, and nociceptive input, shapes afferent conditions within the nervous system. These inputs contribute to Persistent Afferent Bias (PAB), characterized by altered precision weighting of afferent signals and state-dependent processing within distributed neural networks. Under sustained conditions, such bias leads to a shift in autonomic regulation conceptualized as Somato-Psychic Autonomic Dysregulation (SPAD), reflected in altered regulatory set-points and reduced flexibility. This dysregulated state may give rise to functional manifestations, including behavioral, emotional, and cognitive symptoms, conceptualized as Somato-Psychic Syndrome (SPS). The cascade is inherently directional, consistent with the principle of Directional Developmental Sequencing (DDS), and reflects the hierarchical organization of brain–body regulatory processes. The upper and lower panels indicate that these processes occur within state-dependent neural regulation and distributed neural systems spanning sensory, limbic, autonomic, and cognitive networks. For clarity, the figure emphasizes the primary directional organization of the proposed cascade. Reciprocal and state-dependent interactions among levels of organization are discussed in the text but are not explicitly represented in the diagram.

The hierarchical organization of these processes is schematically illustrated in Figure 1.

## 4 Relationship to existing models

The relationship between altered afferent input, autonomic regulation, and higher-order functional outcomes has been examined across multiple disciplines, including sensorimotor neuroscience, pain science, autonomic physiology, predictive processing, and stress biology. Together, these fields have generated a substantial body of evidence demonstrating that sensory input influences neural processing, autonomic regulation, and behavioral adaptation.

Across these disciplines, several complementary frameworks have emerged to explain specific aspects of the interaction between bodily input and regulatory function. These include sensorimotor integration and fusimotor control, somato-autonomic reflexes, central sensitization and nociplastic pain, neurovisceral integration, predictive processing, and allostasis. Each framework provides valuable insights into specific mechanisms by which afferent signals shape physiological and behavioral outcomes.

Taken together, these frameworks may be viewed as describing different components of a broader regulatory architecture. Importantly, they are largely compatible rather than mutually exclusive. However, they have evolved within separate disciplinary traditions, employ different conceptual languages, and focus on different levels of biological organization. As a result, interactions among these mechanisms are often discussed primarily within the boundaries of individual fields, while their potential integration into a unified regulatory framework remains limited. In some instances, competing explanatory emphases have contributed to conceptual fragmentation despite substantial overlap in the underlying physiological processes being described.

The present framework builds upon these established lines of research and examines whether these seemingly distinct mechanisms can be understood as interconnected elements of a hierarchically organized regulatory process. Within this perspective, Persistent Afferent Bias (PAB) is proposed as an integrative construct that links established mechanisms of afferent modulation, autonomic regulation, and state-dependent processing into a coherent regulatory cascade extending from somatic disruption to higher-order functional manifestations.

The following sections briefly review each framework’s contribution and clarify its relationship to the present model.

### 4.1 Sensorimotor integration and Fusimotor control

Contemporary sensorimotor neuroscience has demonstrated that afferent input is not a fixed representation of peripheral events but is continuously modulated by central and peripheral regulatory processes. In particular, models of sensorimotor integration and fusimotor control have shown that muscle spindle sensitivity is dynamically regulated through interactions between alpha and gamma motor systems, enabling the nervous system to adjust proprioceptive gain according to behavioral context and task demands (Lan, 2012; Li et al., 2015).

Within this framework, proprioceptive information is understood as an actively regulated component of motor control rather than a passive sensory signal. Descending influences continuously shape spindle sensitivity, reflex responsiveness, and sensorimotor integration, allowing the organism to adapt posture and movement to changing environmental and physiological conditions. These findings strongly support the principle that afferent weighting is state-dependent and subject to ongoing modulation.

However, the primary focus of these models remains the regulation of posture, movement, and motor adaptation. While they provide a detailed account of how proprioceptive gain is adjusted within sensorimotor systems, they do not explicitly address how persistent alterations in afferent weighting may become embedded within broader autonomic regulatory states or contribute to higher-order functional manifestations.

Within the present framework, these mechanisms are regarded as an important component of the processes underlying Persistent Afferent Bias (PAB). Rather than replacing existing models of sensorimotor integration, PAB incorporates their insights into a broader regulatory perspective in which altered afferent weighting may influence not only motor control but also autonomic regulation and its downstream functional consequences.

### 4.2 Somato-autonomic reflexes

The influence of somatic afferent input on autonomic regulation has long been recognized within the framework of somato-autonomic reflex physiology. Experimental studies have demonstrated that stimulation of somatic tissues can influence cardiovascular, gastrointestinal, and other autonomic functions through reflex pathways involving spinal, brainstem, and supraspinal mechanisms (Sato and Schmidt, 1987). More recent work has further supported the close functional interaction between afferent signaling, autonomic regulation, and distributed regulatory networks, emphasizing that autonomic responses emerge from dynamic integration across multiple physiological systems rather than from isolated reflex pathways alone (Wang and Chang, 2024; Daibes et al., 2025).

Within this framework, somatic afferent signals are not limited to sensorimotor processing but also contribute to the regulation of visceral and autonomic functions. These findings provide important evidence that bodily input can influence autonomic state through identifiable neurophysiological pathways, thereby supporting the broader principle that somatic and autonomic systems operate as functionally interconnected components of a larger regulatory network.

A key distinction between classical somato-autonomic reflex models and the present framework concerns timescale and regulatory persistence. Somato-autonomic reflexes primarily describe transient physiological responses to somatic stimulation. In contrast, Persistent Afferent Bias (PAB) is proposed as a state-dependent shift in regulatory organization, in which repeated or sustained afferent influences contribute to a more stable alteration of autonomic set-point and regulatory flexibility. In this sense, PAB refers not to a reflex response itself, but to the progressive embedding of afferent influences within the organism’s regulatory state.

This distinction parallels broader developments in contemporary neuroscience, where increasing attention has shifted from isolated stimulus–response relationships toward mechanisms of state-dependent regulation, network adaptation, and regulatory load. From this perspective, individual somato-autonomic reflexes may be viewed as short-term expressions of processes that, when repeatedly activated over time, contribute to more persistent patterns of autonomic organization.

Within the present framework, somato-autonomic reflexes are therefore regarded as one of several mechanisms through which altered afferent input may influence autonomic regulation. Persistent Afferent Bias extends this perspective by examining how repeated or sustained afferent influences may become incorporated into longer-term regulatory configurations, thereby linking somatic input not only to immediate autonomic responses but also to broader patterns of physiological and functional adaptation.

### 4.3 Central sensitization and Nociplastic pain

Contemporary pain science has provided important insights into the ways sustained afferent input can alter neural processing and regulatory function. In particular, the concepts of central sensitization and nociplastic pain have contributed substantially to understanding how persistent nociceptive signaling may influence perception, behavior, motor control, and physiological regulation (Woolf, 2011; Kosek et al., 2016; Raja et al., 2020). These frameworks emphasize that alterations in neural responsiveness may persist beyond the immediate presence of tissue injury and can become embedded within broader patterns of physiological adaptation.

Central sensitization refers to increased responsiveness within central nociceptive pathways, resulting in amplification of sensory processing and altered responses to incoming stimuli (Woolf, 2011). Closely related concepts within nociplastic pain research further emphasize that clinically significant alterations in pain processing may occur even in the absence of clear structural pathology, highlighting the importance of central regulatory mechanisms in shaping symptom expression (Kosek et al., 2016; Raja et al., 2020). These models provide compelling evidence that sustained afferent input can contribute to long-term changes in neural processing and regulatory function.

Related perspectives have also emphasized that persistent pain is associated with adaptive reorganization across multiple regulatory levels. In their influential theory of pain adaptation, Hodges and Tucker (2011) proposed that pain-related changes in motor behavior may initially serve a protective function but can become persistent and maladaptive over time. Their model provides an important example of how sustained somatic input may contribute to long-term changes in neural and behavioral regulation. The present PAB framework is broadly compatible with this perspective but extends it beyond nociceptive mechanisms to encompass proprioceptive, interoceptive, and other afferent influences, as well as their potentially cumulative and mutually reinforcing contributions to autonomic regulatory load. From this perspective, persistent afferent conditions may collectively impose long-term demands that gradually exceed the adaptive capacity of autonomic regulation, thereby contributing to the emergence and maintenance of dysregulated regulatory states.

Clinical observations from myofascial pain research provide another relevant example of how localized somatic disturbances may be associated with wider regulatory consequences. In their review of myofascial trigger point mechanisms, Bron and Dommerholt (2012) described how persistent nociceptive input arising from localized musculoskeletal sources may be associated not only with sensory manifestations, but also with motor and autonomic changes. Although their work focuses specifically on myofascial pain, it illustrates the broader principle that sustained somatic influences may extend beyond local tissue effects and contribute to wider patterns of physiological regulation. Within the present framework, such observations are compatible with the concept of Persistent Afferent Bias and may represent one example of how localized somatic conditions contribute to longer-term regulatory adaptation and downstream functional consequences.

An additional distinction relevant to the present framework concerns the relationship between nociception and pain. Contemporary pain science recognizes that nociceptive signaling and the conscious experience of pain are not identical phenomena (Raja et al., 2020). While pain represents a conscious perceptual and affective interpretation of nociceptive activity, nociceptive signaling itself may occur without reaching the threshold of conscious awareness. Consequently, the absence of pain does not necessarily imply the absence of ongoing nociceptive signaling.

Within this context, the present framework places particular emphasis on persistent low-grade and persistent subthreshold nociceptive input. Such input may not generate clinically prominent pain yet may continue to influence autonomic regulation, neural gain, and state-dependent processing over extended periods of time. Similar considerations have been proposed in relation to persistent low-grade nociceptive drive and its potential regulatory consequences within the Somato-Psychic Pathway framework (Živný, 2026).

A further distinction concerns the range of afferent channels involved. Central sensitization and nociplastic pain primarily focus on nociceptive mechanisms and pain-related outcomes. In contrast, Persistent Afferent Bias (PAB) is proposed as a broader regulatory construct that incorporates not only nociceptive influences but also altered proprioceptive, interoceptive, and other afferent inputs that may contribute to long-term changes in the regulatory state. From this perspective, nociceptive signaling represents one of several afferent channels through which persistent alterations in autonomic regulation and sensory weighting may emerge.

This distinction broadens the discussion beyond pain perception itself and highlights the potential regulatory significance of persistent afferent input even in the absence of conscious pain (Fields, 2004; Woolf, 2011; Živný, 2026). Within the present framework, central sensitization and nociplastic pain are therefore regarded as important contributors to understanding how sustained afferent influences may alter neural processing. Persistent Afferent Bias builds upon these insights by examining how such influences, whether nociceptive or non-nociceptive, may become integrated into longer-term patterns of autonomic regulation and higher-order functional organization. In this sense, central sensitization and Persistent Afferent Bias may be viewed as partially overlapping and potentially complementary constructs operating at different levels of analysis. While central sensitization primarily describes altered responsiveness within nociceptive systems, Persistent Afferent Bias addresses a broader range of afferent influences and their potential contribution to autonomic regulation and state-dependent organization.

### 4.4 Neurovisceral integration

The Neurovisceral Integration model has provided one of the most influential contemporary frameworks for understanding the relationship between autonomic regulation, emotional functioning, and higher-order cognitive control (Thayer and Lane, 2000; Thayer et al., 2012). Central to this framework is the concept of the Central Autonomic Network (CAN), a distributed system of cortical and subcortical structures that coordinates autonomic regulation while simultaneously supporting attention, emotional regulation, executive function, and adaptive behavior. Subsequent work has extended these principles to emotional dysregulation and psychopathology, highlighting autonomic flexibility as a transdiagnostic marker of regulatory capacity (Beauchaine, 2015).

Related work on interoception and body–brain integration has further emphasized the importance of visceral signaling in shaping emotional experience, self-regulation, and higher-order cognitive processes (Craig, 2002; Critchley and Harrison, 2013). Together, these perspectives support the view that autonomic regulation occupies a central position within broader systems of adaptive neural and behavioral organization.

These perspectives collectively support the view that bodily and autonomic states are not merely consequences of higher-order neural processes, but active contributors to emotional experience, cognitive function, and adaptive behavior. From this perspective, autonomic regulation is not only an output of neural activity but also a continuous source of regulatory information that shapes perception, self-regulation, and behavioral adaptation.

Within this model, autonomic flexibility is regarded as a key indicator of regulatory capacity. Measures such as heart rate variability (HRV) are interpreted as reflecting the functional integrity of neural networks involved in self-regulation, cognitive flexibility, and adaptive responses to environmental demands (Thayer et al., 2012; Beauchaine, 2015). Reduced autonomic flexibility has been associated with a broad range of emotional, behavioral, and neurodevelopmental difficulties, supporting the view that autonomic regulation represents a central component of higher-order functioning.

Related perspectives, including Polyvagal Theory, have further emphasized the importance of autonomic state in shaping emotional regulation, social engagement, and adaptive behavioral responses (Porges, 2007, 2025, 2026). Although these models differ in their theoretical emphasis and explanatory scope, they converge in highlighting autonomic regulation as a fundamental determinant of emotional and cognitive functioning.

The Neurovisceral Integration model further emphasizes the reciprocal nature of interactions between autonomic state and cognitive-emotional processes. Changes in autonomic regulation influence perception, attention, and behavior, while cognitive appraisal, emotional experience, and top-down regulatory processes, in turn, influence autonomic function (Thayer and Lane, 2000; Critchley and Harrison, 2013). This bidirectional perspective has become a foundational principle in contemporary psychophysiology and affective neuroscience.

The present framework is fully compatible with these principles. However, its primary focus differs from that of Neurovisceral Integration. Whereas Neurovisceral Integration provides a sophisticated account of how autonomic regulation interacts with emotional and cognitive processes, Persistent Afferent Bias (PAB) focuses primarily on the afferent conditions that may contribute to the emergence and maintenance of altered autonomic states.

Within this perspective, persistent alterations in proprioceptive, interoceptive, nociceptive, or other afferent inputs may represent one of the possible pathways through which autonomic regulatory states emerge, become stabilized, and subsequently influence higher-order functional domains. The emphasis is therefore placed not primarily on the consequences of altered autonomic regulation, but on the afferent influences that may contribute to its development and persistence.

In this sense, Neurovisceral Integration and Persistent Afferent Bias may be viewed as complementary rather than competing frameworks. Neurovisceral Integration provides a detailed description of the relationship between autonomic regulation and higher-order functions, whereas Persistent Afferent Bias seeks to clarify how sustained alterations in afferent conditions may contribute to establishing the autonomic states on which these relationships depend.

### 4.5 Predictive processing and interoceptive models

Predictive Processing has emerged as one of the most influential contemporary frameworks for understanding perception, action, and adaptive regulation. Within this perspective, the nervous system is viewed as a predictive organ that continuously generates models of the body and environment, compares incoming sensory information with these predictions, and updates its internal representations in order to minimize prediction error (Friston, 2010). Perception is therefore understood not as a passive registration of sensory events but as an active process of inference shaped by both incoming signals and prior expectations.

Related models of interoceptive inference have extended these principles to the regulation and perception of bodily states. Interoceptive signals are not simply monitored but are continuously interpreted within predictive frameworks that contribute to emotional experience, self-awareness, and adaptive behavior (Craig, 2002; Barrett and Simmons, 2015; Seth and Friston, 2016). From this perspective, bodily signals play an active role in shaping perception, cognition, and affect, rather than serving merely as downstream consequences of higher-order neural activity.

These frameworks provide a powerful account of how sensory information acquires meaning within neural systems. In particular, they highlight the importance of sensory weighting, prediction error minimization, and context-dependent processing. The present framework is fully compatible with these principles and shares the assumption that neural responses are shaped not only by incoming sensory signals but also by the organism’s regulatory state.

However, the primary focus of Predictive Processing concerns how sensory information is interpreted, weighted, and incorporated into internal models. Persistent Afferent Bias (PAB) focuses more specifically on the afferent conditions that may contribute to the emergence of persistent weighting biases within these predictive systems. From this perspective, prolonged alterations in proprioceptive, interoceptive, nociceptive, or other afferent inputs may influence the sensory landscape upon which predictive and interoceptive processes operate.

Within this framework, Persistent Afferent Bias may be understood as a state-dependent alteration in afferent weighting that influences the balance of information available to predictive systems. Rather than competing with Predictive Processing, PAB may be viewed as one of the possible pathways through which persistent afferent conditions contribute to the formation, stabilization, and maintenance of altered regulatory states.

Within the proposed regulatory cascade, Predictive Processing and interoceptive models primarily describe processes at the levels of interpretation, prediction, and representation, whereas Persistent Afferent Bias addresses conditions arising earlier in the sequence, at the levels of afferent organization and sensory weighting. The present framework, therefore, focuses not on how predictive systems generate interpretations of bodily states, but on how persistent alterations in afferent conditions may influence the informational landscape on which those interpretations are constructed.

In this sense, Predictive Processing and interoceptive models provide a detailed account of how neural systems generate and update representations of bodily and environmental states, whereas Persistent Afferent Bias seeks to clarify how sustained alterations in afferent input may contribute to the conditions under which such predictive processes operate. The two perspectives are therefore complementary and may be viewed as addressing different levels of the same regulatory architecture.

### 4.6 Allostasis and regulatory load

The concepts of allostasis and allostatic load have provided an influential framework for understanding how physiological systems adapt to changing environmental and internal demands (McEwen, 1998, 2007; McEwen and Gianaros, 2011; Sterling, 2012; Guidi et al., 2021). In contrast to classical homeostatic models, allostasis emphasizes the dynamic adjustment of regulatory processes to maintain functional stability under varying conditions. From this perspective, physiological regulation is understood as an adaptive and continuously evolving process rather than a return to a fixed equilibrium.

Within this framework, repeated or prolonged exposure to regulatory demands may result in allostatic load, reflecting the cumulative physiological burden associated with sustained adaptation. Such processes have been linked to alterations in autonomic regulation, neuroendocrine activity, emotional functioning, cognitive performance, and long-term health outcomes (McEwen and Gianaros, 2011; Guidi et al., 2021; Sterling, 2012). Contemporary models further emphasize that adaptive regulation is not merely a response to stress but a fundamental organizing principle of physiological function across the lifespan (Sterling, 2012).

These concepts provide an important account of how regulatory systems change over time in response to persistent challenges. In particular, they help explain the emergence of altered regulatory set-points, reduced flexibility, and the stabilization of physiological states that may initially arise as adaptive responses but later become maladaptive, concepts that were central to the original formulation of allostatic load (McEwen, 1998). From a systems perspective, multiple sources of physiological demand may interact and reinforce one another through shared neural, autonomic, endocrine, and immune pathways, thereby contributing to cumulative regulatory burden (Kotas and Medzhitov, 2015; Guidi et al., 2021).

The present PAB framework is fully compatible with these principles. It does not propose a new form of allostatic adaptation, but rather examines whether persistent afferent conditions may represent one specific pathway contributing to the regulatory demands described within the allostatic load framework (McEwen, 1998). However, it focuses on a different segment of the regulatory sequence. Whereas allostasis primarily describes the adaptive consequences of prolonged regulatory demands and the physiological processes that follow, Persistent Afferent Bias (PAB) focuses on one of the possible sources of such demands. Specifically, persistent alterations in proprioceptive, interoceptive, nociceptive, or other afferent inputs may contribute to the regulatory burden that subsequently requires allostatic adaptation.

A key distinction of the present framework is reflected in the term Persistent Afferent Bias itself. While many contemporary models describe mechanisms of sensory modulation, autonomic adaptation, predictive regulation, or allostatic adjustment, the present framework places particular emphasis on persistence as a defining characteristic of the underlying afferent condition. The concept does not refer to transient fluctuations in sensory weighting but to chronic alterations in afferent input that remain active for sufficient periods to contribute to the emergence, stabilization, and maintenance of a persistently altered autonomic regulatory state.

Within the proposed regulatory cascade, allostasis primarily describes adaptive responses and physiological consequences that arise from sustained regulatory demands, whereas Persistent Afferent Bias addresses conditions that arise earlier in the sequence, at the level of afferent organization and sensory weighting. From this perspective, the persistence of the afferent condition enables the emergence of more stable regulatory adaptations, including shifts in autonomic set points and reductions in regulatory flexibility.

Importantly, these adaptations are not regarded as permanent. Rather, they are understood as state-dependent and potentially reversible responses to chronic regulatory demand. However, they are expected to persist for as long as the primary source of afferent bias remains active. Resolution of the underlying afferent driver—whether through spontaneous recovery, developmental reorganization, or appropriately targeted intervention at the origin of the disturbance—may reduce ongoing regulatory demand and facilitate the restoration of autonomic flexibility and more adaptive regulatory states.

Within this perspective, Persistent Afferent Bias may be viewed as one of the possible pathways through which sustained afferent conditions contribute to allostatic load and the emergence of altered autonomic states. In this sense, allostasis and Persistent Afferent Bias are not competing explanations but rather complementary frameworks that address different stages of the same regulatory process. Allostasis explains how the organism adapts to prolonged demands, whereas Persistent Afferent Bias seeks to clarify how specific chronic afferent conditions may contribute to the generation, maintenance, and persistence of those demands.

### 4.7 Distinctive features of Persistent Afferent Bias

The preceding frameworks collectively provide important insights into the relationships among sensory processing, autonomic regulation, physiological adaptation, and higher-order functioning. Sensorimotor integration models explain dynamic modulation of proprioceptive gain; somato-autonomic reflex models describe pathways linking bodily input and autonomic responses; central sensitization and nociplastic pain models account for persistent alterations in nociceptive processing; Neurovisceral Integration characterizes relationships between autonomic regulation and higher-order functions; Predictive Processing explains how sensory information is interpreted within predictive systems; and allostasis describes adaptive responses to prolonged regulatory demands.

Within this perspective, Persistent Afferent Bias (PAB) is introduced as an integrative construct focused specifically on the organization, weighting, and persistence of afferent conditions that may contribute to the emergence and maintenance of altered autonomic states. Rather than representing an alternative to existing frameworks, it proposes that sensorimotor integration, somato-autonomic reflexes, central sensitization, Neurovisceral Integration, Predictive Processing, and allostasis may describe different components of a broader regulatory sequence. The conceptual definitions and hierarchical positions of the core constructs within this proposed regulatory cascade (Somatic Disruption → DPI → PAB → SPAD → SPS) are summarized in Table 1.

**Table 1:** Core constructs within the proposed regulatory cascade (somatic disruption → altered afferent conditions [e.g., DPI] → PAB → SPAD → SPS).

Construct	Hierarchical position	Working definition	Candidate markers	Current assessment approaches
Somatic Disruption (SD)	Etiological source	Disturbance of somatic structural–functional integrity capable of generating abnormal afferent input and/or direct peripheral influences on autonomic regulation.	Axial asymmetry, segmental restriction, postural instability, altered movement patterns, musculoskeletal dysfunction.	Clinical structural and functional examination; postural assessment; movement analysis.
Distorted Proprioceptive Information (DPI)	Afferent disturbance	Mismatch between peripheral somatic input and central predictive models of posture and movement, resulting in reduced proprioceptive coherence.	Postural asymmetry, impaired sensorimotor integration, altered body schema, proprioceptive inconsistency.	Clinical sensorimotor assessment; postural testing; proprioceptive tasks; experimental sensorimotor paradigms.
Persistent Afferent Bias (PAB)	Regulatory mechanism	Persistent state-dependent alteration in afferent weighting whereby selected afferent streams exert disproportionate influence on neural and autonomic regulation.	Altered sensory weighting, persistent regulatory bias, and state-dependent shifts in afferent influence.	Currently inferred indirectly from clinical and experimental observations; future multimodal physiological and computational approaches are required.
Somato-Psychic Autonomic Dysregulation (SPAD)	Autonomic regulatory state	Persistent reduction in autonomic flexibility arising from sustained somatic regulatory load and biased afferent conditions.	Reduced autonomic adaptability, altered stress recovery, autonomic instability, and dysregulated physiological state.	Clinical autonomic assessment; HRV, pupillometry, baroreflex sensitivity, autonomic reactivity measures (where available).
Somato-Psychic Syndrome (SPS)	Clinical phenotype	Clinical phenotype emerging secondary to persistent SPAD and characterized by emotional, behavioral, cognitive, developmental, and regulatory manifestations.	Sleep dysregulation, emotional lability, anxiety features, attentional instability, behavioral variability, stress intolerance.	Clinical assessment, behavioral observation, and standardized psychological and neuropsychological evaluation.

A central feature of the present framework is the emphasis on persistence. While many existing models describe transient regulatory processes, adaptive responses, or mechanisms of sensory modulation, Persistent Afferent Bias specifically addresses chronic alterations in afferent input that remain active for sufficient periods to contribute to the emergence, stabilization, and maintenance of persistently altered autonomic regulatory states. In this sense, persistence is not merely a descriptive feature of the model but one of its primary explanatory variables.

A second distinguishing feature concerns the level of analysis. Many contemporary frameworks primarily address processes occurring at the level of interpretation, adaptation, or downstream consequences. In contrast, PAB focuses on afferent conditions that arise earlier in the proposed regulatory sequence, examining how persistent alterations in proprioceptive, interoceptive, nociceptive, and other afferent inputs may influence the regulatory landscape on which higher-order processes subsequently operate.

The concept of Persistent Afferent Bias emerged from consistent clinical observations in pediatric and adolescent patients treated for Somato-Psychic Syndrome (SPS) (Živný, 2026). These observations repeatedly suggested that modification of presumed somatic drivers may be followed by observable changes across hierarchically higher regulatory domains, including autonomic regulation, emotional and behavioral functioning, cognitive performance, developmental trajectories, and other neuropsychologically testable domains. In a substantial proportion of these cases, the presumed source of persistent afferent input was a clinically diagnosable axial somatic disruption affecting the postural system, which could subsequently be addressed therapeutically.

Importantly, the clinical observations that informed the development of the PAB concept preceded its formal theoretical formulation. The observations were therefore derived from recurring clinical phenomena rather than from prior classification according to the PAB construct itself.

These observations are exceptional not because of their rarity, but because they provide a particularly informative natural clinical model. In such cases, a clinically diagnosable axial somatic disruption affecting the postural system may serve as a putative source of persistent afferent input that is also therapeutically modifiable. This creates an unusual opportunity to observe whether changes occurring at the level of afferent organization are followed by corresponding changes across higher levels of the proposed regulatory hierarchy. Similar clinical observations have previously informed the formulation of the SPS and SPAD constructs and have been reported in preliminary form in the clinical neuropsychology literature (Zivna and Zivny, 2025).

An additional feature of this model is the relative temporal proximity between intervention and observable change. Unlike many developmental, psychiatric, or chronic disease models, the proposed sequence can often be observed within clinically relevant time frames, allowing changes across multiple regulatory levels to be followed without prolonged longitudinal observation. Changes in higher regulatory domains often become apparent within a comparatively short time frame, permitting observation of regulatory dynamics in near real time. This provides a particularly informative context for generating and refining hypotheses concerning hierarchical relationships among afferent organization, autonomic regulation, and higher-order functional outcomes.

Within the framework of the Somato-Psychic Pathway (SPP) and Directional Developmental Sequencing (DDS) (Živný, 2026), the explanatory value of this clinical model lies not in proving causality, but in providing a coherent observational framework through which the proposed relationships can be examined and further tested.

The present framework, therefore, positions PAB as a complementary rather than competing account of regulatory organization. Its principal contribution lies in emphasizing persistent afferent conditions as a potential source of long-term regulatory adaptation and in providing a conceptual bridge linking somatic disruption, autonomic regulation, and higher-order functional manifestations within a single hierarchical model.

## 5 From Persistent Afferent Bias to autonomic dysregulation

Persistent Afferent Bias represents more than a transient shift in sensory weighting. Under conditions of sustained input, it may lead to a progressive reorganization of regulatory dynamics within the nervous system, particularly at the level of autonomic control.

At the spinal and brainstem levels, continuous afferent input—whether nociceptive, proprioceptive, or interoceptive—can alter the balance between excitatory and inhibitory influences within local circuits. Such input may contribute to dorsal horn sensitization and to changes in descending modulatory systems, including structures within the periaqueductal gray and rostral ventromedial medulla. These processes influence not only pain perception but also broader aspects of autonomic regulation.

At higher levels of integration, afferent bias may affect the activity of networks involved in salience detection and interoceptive processing, particularly within the insular cortex and anterior cingulate cortex. Through these pathways, altered sensory weighting may contribute to changes in autonomic tone, behavioral responses, and the subjective experience of bodily states.

Crucially, these changes do not necessarily reflect structural pathology but rather a shift in the system’s regulatory set-point. When sustained over time, such shifts may become functionally embedded, leading to reduced autonomic flexibility and a tendency toward biased physiological responses.

Within this context, Somato-Psychic Autonomic Dysregulation (SPAD) may be understood as a stabilized manifestation of Persistent Afferent Bias at the level of autonomic regulation. While afferent bias itself reflects a dynamic and potentially reversible state, SPAD represents a more enduring configuration of the system, characterized by altered baseline regulation and reduced adaptive capacity.

This formulation suggests that interventions targeting neural gain or autonomic tone alone may be insufficient if the primary source of biased afferent input remains unaddressed. In contrast, therapeutic approaches that reduce or normalize the underlying afferent drive—particularly within the somatic system—may facilitate a more stable reorganization of regulatory function.

## 6 Testable predictions

The proposed framework generates several empirically testable predictions regarding the relationship between somatic input, afferent bias, autonomic regulation, and higher-order functional manifestations.

First, if Persistent Afferent Bias (PAB) contributes to shifts in autonomic state, then targeted modification of somatic drivers should produce measurable changes in autonomic markers. Such changes may be detectable using indices of autonomic function, including heart rate variability (HRV), pupillary dynamics, baroreflex sensitivity, or respiratory variability. Importantly, these effects are expected to occur even in the absence of direct central interventions, reflecting the bottom-up influence of somatic input on the regulatory state.

Second, the model predicts that the clinical effects of somatic interventions should follow a characteristic temporal sequence. Changes in autonomic regulation—such as improved physiological stability, increased autonomic flexibility, or reduced baseline arousal—should precede observable changes in emotional, behavioral, cognitive, or developmental domains. This sequence reflects the hierarchical organization of regulatory systems and is consistent with the principle of Directional Developmental Sequencing (DDS).

Third, Persistent Afferent Bias is expected to be context-sensitive. The same somatic stimulus may elicit different physiological or behavioral responses depending on the organism’s current regulatory state. This prediction aligns with the concept of state-dependent processing and suggests that variability in clinical response is not random but reflects underlying differences in system state.

Fourth, if somatic sources of persistent afferent input remain unaddressed, interventions targeting neural gain, autonomic tone, or higher-order manifestations alone are predicted to produce only transient effects. In such cases, the system may gradually revert toward its previous regulatory configuration due to the continued presence of a Persistent Afferent Bias. Conversely, interventions that effectively reduce or normalize primary somatic drivers are predicted to be associated with more stable and sustained changes in autonomic regulation and downstream functional outcomes.

Fifth, the model predicts that different sources of afferent input—including nociceptive, proprioceptive, and interoceptive pathways—may contribute to Persistent Afferent Bias through partially overlapping but distinguishable mechanisms. This raises the possibility of identifying physiologically and clinically distinct subtypes of afferent bias characterized by different regulatory signatures and patterns of manifestation.

These predictions also provide criteria by which the proposed framework may be evaluated. Findings that would support the model include demonstration of measurable relationships between somatic disruption, autonomic regulation, and higher-order functional manifestations; observation of the predicted temporal sequence in which autonomic changes precede emotional, behavioral, cognitive, or developmental improvements; and evidence that modification of persistent afferent conditions is associated with corresponding changes in autonomic flexibility and downstream functional outcomes.

Conversely, the framework would be weakened if autonomic changes consistently failed to precede higher-order functional changes, if modification of presumed somatic drivers showed no relationship to autonomic regulation, or if the proposed hierarchical sequence could not be demonstrated across independent clinical populations.

More fundamentally, the model would be challenged if persistent afferent conditions were shown to have no systematic relationship to autonomic regulation, or if emotional, behavioral, cognitive, and developmental manifestations consistently occurred independently of measurable changes in autonomic state. Such findings would argue against the central role proposed for Persistent Afferent Bias within the present framework and would require substantial revision of the model.

Importantly, the present framework is not intended to explain all forms of psychopathology. Rather, it proposes one specific, but likely not uncommon, pathophysiological pathway through which somatic disruption may contribute to autonomic dysregulation and subsequently to a subset of emotional, behavioral, cognitive, and developmental manifestations. Although this pathway may account for a clinically meaningful proportion of cases, it is unlikely to represent more than one of several major etiopathogenetic routes leading to psychopathology. Other pathways—including primary genetic, neurodevelopmental, neuropsychiatric, inflammatory, environmental, and psychosocial mechanisms—remain fully compatible with the present framework but are not the subject of the present model.

## 7 Distributed sensorimotor networks

The reinterpretation of facilitation reflects a broader shift toward understanding sensorimotor regulation as a property of distributed neural networks rather than isolated spinal segments (Bassett and Sporns, 2017; Sporns, 2011). These networks integrate information across multiple levels of the nervous system, including spinal interneurons, brainstem nuclei, cerebellar circuits, cortical sensorimotor areas, and peripheral autonomic structures (Shadmehr and Krakauer, 2008).

Within this framework, alterations in afferent weighting are not confined to discrete anatomical regions but emerge from interactions across the network as a whole.

Accordingly, the classical spinal segment may be more appropriately understood as a functional node within a larger regulatory system, rather than as an independent physiological unit.

## 8 Plastic but reversible neural states

A key concept in contemporary neuroscience is the distinction between structural change and state-dependent change. Structural changes involve anatomical alterations and tissue remodeling, whereas state changes reflect dynamic shifts in neural gain, reflex thresholds, autonomic balance, and sensorimotor integration.

Such state-dependent changes are inherently plastic yet potentially reversible, reflecting ongoing modulation within neural systems rather than permanent structural transformation (Fields, 2004; Woolf, 2011).

This distinction provides a plausible explanation for the rapid clinical improvements often observed following interventions targeting the musculoskeletal system and related neuromodulatory mechanisms. In this context, therapeutic effects are more appropriately understood as the modulation of the system state rather than as direct structural correction.

## 9 Limitations of the musculoskeletal model

While the reinterpretation of osteopathic facilitation as Persistent Afferent Bias represents an important conceptual advance, explanations restricted primarily to musculoskeletal and pain-related mechanisms remain insufficient to account for the broader regulatory architecture proposed in the present framework.

Although such models provide valuable insight into altered sensorimotor processing and nociceptive influences on neural regulation, they do not fully explain the emergence, persistence, and higher-order consequences of altered afferent conditions. In particular, they provide only limited accounts of long-term autonomic dysregulation, hierarchical developmental processes, and the emotional, behavioral, cognitive, and developmental manifestations that may arise when altered afferent input becomes functionally embedded within the organism’s regulatory state.

The present framework, therefore, proposes a broader regulatory perspective in which musculoskeletal, proprioceptive, interoceptive, nociceptive, autonomic, and higher-order functional processes are viewed as interconnected components of a hierarchical regulatory cascade. Within this perspective, musculoskeletal mechanisms remain important contributors, but they represent only one particular source of persistent afferent conditions within a larger regulatory system linking somatic disruption, autonomic regulation, and downstream functional outcomes.

Accordingly, further conceptual expansion is required to situate these mechanisms within an integrative framework that incorporates somatic, autonomic, developmental, emotional, behavioral, and cognitive levels of organization (Nijs and Van Houdenhove, 2009).

## 10 The axial postural system as a major source of afferent Bias

A critical element that remains underrepresented in contemporary interpretations is the role of the axial postural system as a source of afferent input influencing neural regulation.

The axial system, comprising the spine, deep stabilizing musculature, ligamentous structures, and associated proprioceptive networks, represents a major source of continuous sensory input to the central nervous system. This input contributes to the ongoing calibration of sensorimotor integration, autonomic tone, and overall neural state (Langevin, 2006; Langevin et al., 2013).

Disturbances within this system, whether structural or functional, can lead to persistent alterations in proprioceptive signaling. Such alterations may influence spinal reflex excitability, brainstem autonomic regulation, cerebellar processing, and cortical sensorimotor representations.

Within this context, Persistent Afferent Bias (PAB) may arise not only from nociceptive or peripheral sources but also from altered proprioceptive input originating within the axial system itself. This perspective provides a mechanistic link between classical osteopathic palpatory findings and contemporary models of distributed neural regulation.

Given the density and regulatory significance of axial proprioceptive input, disturbances within this system may be among the most significant sources of Persistent Afferent Bias, rather than merely one of several contributing factors.

## 11 The Somato-Psychic Pathway

The Somato-Psychic Pathway (SPP), as previously described by Živný (2026), provides a conceptual framework linking somatic disturbances to autonomic regulation and higher-order mental functions.

Within this model, disturbances within the somatic system lead to altered proprioceptive and interoceptive input (Craig, 2009), which in turn shapes afferent conditions influencing neural gain and regulatory state. At the level of neural processing, these changes may manifest as Persistent Afferent Bias (PAB), reflecting a state-dependent reorganization of sensory weighting within distributed networks.

Under sustained conditions, such bias contributes to alterations in autonomic regulation conceptualized as Somato-Psychic Autonomic Dysregulation (SPAD). In some cases, this dysregulated state may be associated with broader emotional, behavioral, and cognitive manifestations, conceptualized as Somato-Psychic Syndrome (SPS) (Živný, 2026).

Within this framework, classical concepts such as facilitation are more appropriately understood as intermediate expressions within a broader regulatory cascade rather than as final explanatory endpoints.

In contrast to models that primarily describe the neurophysiological manifestation of altered input, the Somato-Psychic Pathway explicitly addresses the origin and persistence of such bias within the somatic system, thereby shifting the explanatory framework from phenomenology to etiology.

## 12 Directional Developmental Sequencing

Directional Developmental Sequencing (DDS) (Živný, 2026) describes the hierarchical organization of regulatory systems, in which lower-level physiological processes constrain and enable higher-order functions.

Within this framework, somatic structural integrity supports autonomic regulation, which in turn supports sensorimotor integration, emotional regulation, and higher cognitive processes. This organization reflects a directional dependency, in which higher levels of function are contingent upon the stability of underlying regulatory systems.

Accordingly, disturbances at lower levels do not remain localized but propagate across organizational levels, influencing autonomic state, behavioral regulation, and cognitive function. This principle provides a structural basis for understanding how alterations in somatic input and afferent weighting may lead to broader functional consequences.

## 13 Somato-Psychic Autonomic Dysregulation and Somato-Psychic Syndrome

Within this hierarchical framework, Somato-Psychic Autonomic Dysregulation (SPAD) denotes a state of altered autonomic regulation arising from Persistent Afferent Bias (PAB) associated with somatic disruption. In this context, SPAD reflects a shift in the organism’s regulatory set point, characterized by reduced flexibility and an altered autonomic balance.

When such dysregulation becomes sustained, it may give rise to broader functional manifestations, including emotional, behavioral, and cognitive changes, conceptualized as Somato-Psychic Syndrome (SPS). From this perspective, SPS is understood not as a primary disorder, but as a downstream phenotype arising from a persistently dysregulated autonomic state.

Empirical findings further support the functional relevance of autonomic dysregulation in neurodevelopmental and behavioral conditions. Altered sympathetic–parasympathetic balance has been shown to be associated with behavioral dysregulation in children with autism spectrum disorder, linking autonomic function directly to observable clinical manifestations (Fenning et al., 2019). Similarly, atypical patterns of autonomic nervous system activity have been repeatedly reported across neurodevelopmental conditions, including autism and attention-related disorders, suggesting that dysregulation of autonomic state constitutes a transdiagnostic feature rather than a condition-specific anomaly (Owens et al., 2021; Bellato et al., 2020).

Within this context, Somato-Psychic Autonomic Dysregulation (SPAD) may be understood as a clinically relevant expression of such altered regulatory states, providing a mechanistic bridge between somatic disruption, autonomic imbalance, and higher-order functional manifestations.

## 14 Integrating somatic and neuromodulatory interventions within a mechanistic framework

The reinterpretation of facilitation as Persistent Afferent Bias has direct implications for how therapeutic interventions are conceptualized and prioritized. Rather than viewing body-focused and neuromodulatory approaches as separate or competing modalities, this framework situates them within a shared regulatory system governed by hierarchical constraints, in which both act upon different levels of the same underlying process.

Within the Somato-Psychic Pathway (SPP) (Živný, 2026), this distinction reflects a hierarchical organization in which somatic input constrains autonomic regulation, and the autonomic state, in turn, influences higher-order functional domains. From this perspective, interventions may be understood in terms of their position within this hierarchy.

Neuromodulatory interventions primarily influence system state by altering neural gain, autonomic tone, and network-level dynamics. Such approaches may produce rapid and clinically meaningful changes, particularly in conditions characterized by heightened arousal or dysregulated autonomic activity. However, within the present model, these effects are understood as modulation of the system’s current configuration rather than correction of the underlying drivers that sustain Persistent Afferent Bias.

In contrast, body-focused interventions—particularly those targeting sources of somatic disruption—act more directly on the afferent conditions that shape neural processing. By reducing or normalizing biased afferent input, these interventions may alter the system’s input structure, thereby enabling a more stable reorganization of regulatory function.

From this perspective, the distinction between symptomatic modulation and etiological intervention becomes critical. Approaches that primarily target neural gain may provide temporary relief, but are less likely to produce lasting change if the primary sources of afferent bias remain active. Conversely, interventions that effectively address somatic drivers may reduce the need for ongoing modulation by restoring more balanced afferent conditions.

This formulation supports a hierarchical approach to intervention consistent with the organization described within the SPP. Therapeutic strategies may thus be prioritized according to their capacity to influence primary somatic drivers, while neuromodulatory approaches help stabilize the system state and support adaptive reorganization.

In this sense, clinical integration is not merely a matter of combining modalities, but of aligning them within a coherent mechanistic framework that reflects the hierarchical organization of the nervous system.

## 15 Clinical implications: a neurophysiological perspective

The reinterpretation of facilitation as Persistent Afferent Bias (PAB) within distributed neural networks carries important implications for clinical practice. Rather than viewing somatic dysfunction as a primarily structural abnormality, this perspective supports understanding it as a manifestation of an altered regulatory state within the nervous system.

Clinical findings, including palpatory or sensorimotor observations, may be interpreted as localized expressions of network-level bias rather than structural lesions. Accordingly, body-focused and neuromodulatory interventions may be understood as targeted modulation of afferent weighting and autonomic regulation.

The axial postural system serves as a key regulatory interface, and breathing may play an important role in state modulation. This perspective aligns with contemporary views emphasizing the role of autonomic state in shaping perception, behavior, and physiological regulation (Porges, 2025).

From this perspective, neuromodulatory interventions alone may not be sufficient to produce lasting clinical change if the primary source of altered afferent input—namely, somatic disruption—remains unaddressed. While modulation of neural gain and autonomic tone may temporarily improve symptoms, Persistent Afferent Bias may re-establish the previous regulatory state.

The present framework suggests that sustained therapeutic effects may depend on identifying and addressing the underlying primary somatic drivers of altered afferent input. When interventions are appropriately directed toward these primary drivers, they may contribute not only to transient symptom relief but also to a more stable reorganization of regulatory function.

Beyond its mechanistic implications, this framework has direct consequences for clinical reasoning and intervention planning. Rather than focusing primarily on symptomatic modulation or isolated functional deficits, it supports a shift toward identifying and prioritizing primary somatic drivers of altered afferent input. This perspective encourages a more hierarchical and etiologically oriented approach to intervention, in which therapeutic strategies are directed not only at modifying system state but at addressing the underlying sources that sustain Persistent Afferent Bias. In this sense, clinical decision-making becomes less centered on symptom reduction alone and more focused on restoring the conditions necessary for stable regulatory function.

## 16 Conclusion

The transition from the classical facilitated segment model to the concept of Persistent Afferent Bias (PAB) reflects an important evolution in the understanding of how somatic input may influence neural and autonomic regulation. Contemporary neuroscience no longer supports the notion of a persistently hyperexcitable spinal segment as an isolated physiological entity. However, accumulating evidence from sensorimotor neuroscience, pain science, autonomic physiology, predictive processing, neurovisceral integration, and allostasis supports the broader principle that afferent input can exert sustained influences on regulatory state.

Within this context, Persistent Afferent Bias is proposed as a mechanistic construct that describes how chronic alterations in afferent conditions may become functionally embedded in neural and autonomic regulation. A defining feature of the model is its emphasis on persistence. Unlike frameworks that primarily describe transient responses, adaptive consequences, or higher-order manifestations, PAB focuses specifically on the organization, weighting, and persistence of afferent influences that may contribute to the emergence and maintenance of persistently altered autonomic regulatory states.

The Somato-Psychic Pathway (SPP) provides the broader hierarchical framework within which PAB is situated (Živný, 2026). Within this model, somatic disruption may generate multiple forms of altered afferent input, including Distorted Proprioceptive Information (DPI), persistent nociceptive input, interoceptive imbalance, and other afferent influences capable of contributing to Persistent Afferent Bias (PAB). Under sustained conditions, such bias may become functionally embedded within autonomic regulation, contributing to Somato-Psychic Autonomic Dysregulation (SPAD) and, in some cases, to the downstream clinical phenotype of Somato-Psychic Syndrome (SPS). This sequence is consistent with the principle of Directional Developmental Sequencing (DDS) (Živný, 2026), which holds that lower regulatory levels constrain the stability and adaptive capacity of higher-order functions.

The principal contribution of the present work is the proposal that persistent afferent conditions represent a clinically and experimentally relevant mechanism linking somatic disruption to autonomic dysregulation. This formulation provides a conceptual bridge between established neurophysiological models and clinical observations, while generating explicit and falsifiable predictions suitable for future investigation. Within this perspective, sustained changes in regulatory state may depend not only on modulation of neural gain and autonomic tone, but also on the underlying afferent conditions that contribute to their maintenance, a principle broadly consistent with contemporary views on persistent nociceptive drive and state-dependent neural plasticity (Woolf, 2011).

Future research will be necessary to determine the prevalence, boundary conditions, physiological signatures, and clinical relevance of Persistent Afferent Bias, as well as to evaluate its role within broader models of autonomic regulation, neurodevelopment, and mental health.

## 17 Limitations

The Somato-Psychic Pathway is proposed as a conceptual and integrative framework grounded in established neurophysiological principles, rather than as a definitive explanatory model of all clinical phenomena. While the individual components of the model are supported by existing evidence, their integration into a unified hierarchical cascade requires further empirical validation.

In particular, the proposed relationships linking somatic disruption, Persistent Afferent Bias (PAB), Somato-Psychic Autonomic Dysregulation (SPAD), and the higher-order clinical phenotype conceptualized as Somato-Psychic Syndrome (SPS) remain to be systematically investigated in longitudinal and experimental settings.

Future research will be necessary to clarify the specific mechanisms, causal pathways, and clinical applicability of this framework, and to identify potential moderating factors that influence individual variability in regulatory responses.

## Data Availability

The original contributions presented in the study are included in the article/supplementary material, further inquiries can be directed to the corresponding author.
